# Interactions Between Astrocytes and Oligodendroglia in Myelin Development and Related Brain Diseases

**DOI:** 10.1007/s12264-022-00981-z

**Published:** 2022-11-12

**Authors:** Xuelian Hu, Guangdan Yu, Xiang Liao, Lan Xiao

**Affiliations:** 1 School of Medicine, Chongqing University, Chongqing, 400030 China; 2 Department of Histology and Embryology, Chongqing Key Laboratory of Neurobiology, Brain and Intelligence Research Key Laboratory of Chongqing Education Commission, Third Military Medical University, Chongqing, 400038 China

**Keywords:** Astrocyte, Oligodendroglia, Glial network, Myelination, Synaptogenesis, Neuroinflammation

## Abstract

Astrocytes (ASTs) and oligodendroglial lineage cells (OLGs) are major macroglial cells in the central nervous system. ASTs communicate with each other through connexin (Cx) and Cx-based network structures, both of which allow for quick transport of nutrients and signals. Moreover, ASTs interact with OLGs through connexin (Cx)-mediated networks to modulate various physiological processes in the brain. In this article, following a brief description of the infrastructural basis of the glial networks and exocrine factors by which ASTs and OLGs may crosstalk, we focus on recapitulating how the interactions between these two types of glial cells modulate myelination, and how the AST-OLG interactions are involved in protecting the integrity of the blood-brain barrier (BBB) and regulating synaptogenesis and neural activity. Recent studies further suggest that AST-OLG interactions are associated with myelin-related diseases, such as multiple sclerosis. A better understanding of the regulatory mechanisms underlying AST-OLG interactions may inspire the development of novel therapeutic strategies for related brain diseases.

## Introduction

Astrocytes (ASTs) and oligodendrocytes (OLs), the two types of macroglial cells abundant in the central nervous system (CNS), are derived from the same radial glia or neural stem cells. Both were thought to be passive supporters of neurons due to their electric non-excitable characteristic [1]. In recent years, studies have shown that ASTs can transmit information over long distances in the brain by means of the intercellular spread of Ca^2+^ waves [2]. Moreover, ASTs can regulate synaptic development and plasticity through tripartite synapses, and actively participate in brain functions such as learning and memory [3].

Oligodendroglial lineage cells (OLGs) are a series of developing cells that mature progressively from oligodendrocyte precursor cells (OPCs) into post-mitotic myelinating OLs. Besides ensuring the rapid conduction of neuronal action potential by myelin, OLs provide metabolic support to axons through the monocarboxylic acid transporter 1 (MCT1)-mediated lactate shuttle [4]. Recent studies have demonstrated that the proliferation and differentiation of OLGs are highly dynamic and plastic, as the newly-formed myelin sheath is not only necessary for motor learning, but also contributing to the enhancement of fear memory consolidation and synaptic plasticity [5–7]. Moreover, OLGs can adopt an immune phenotype by expressing specific genes previously thought to be unique to immune cells and act as an initiation factor of immune inflammatory diseases such as multiple sclerosis (MS) [8, 9].

**Fig. 1:**
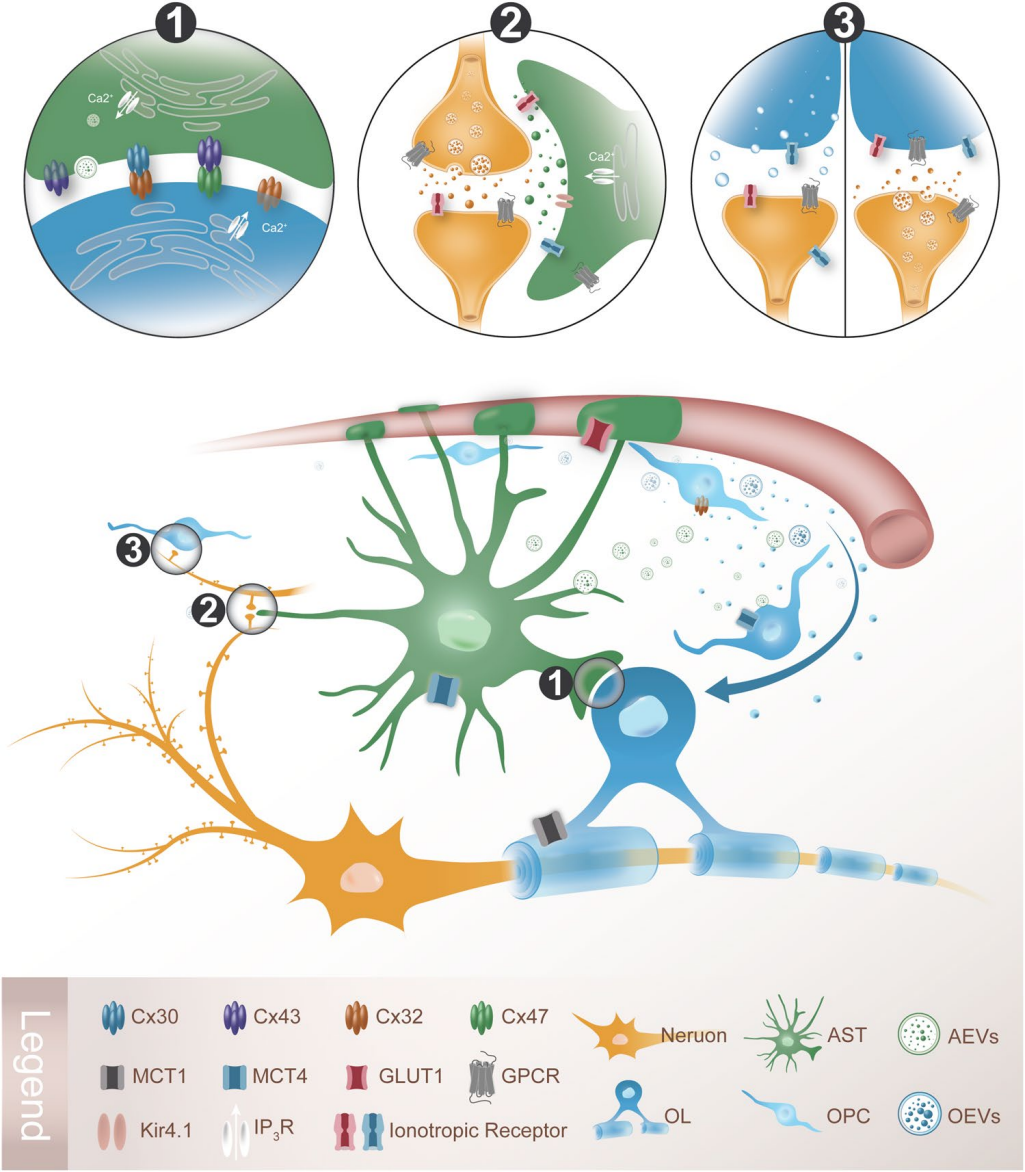
Schematic of AST-OLG interactions at three domains: associated with blood vessels, interacting at the synaptic level, and communicating *via* the Cx-mediated network. ASTs obtain their energy substrate (glucose) from blood vessels through GLUT1 to supply neurons, and the distribution of MCTs among ASTs and OLGs implies a lactate shuttle; OPCs migrate along the blood vessel and differentiate into mature OLs to form myelin; this is regulated by AST secretory factors or AEVs. In the enlarged view: ① ASTs and OLs form heterotypic gap junctions (A:O) or hemichannels by specific Cxs (Cx43/Cx47, Cx30/Cx32) for cell communication and substance exchange. The opening of hemichannels depends on signaling by Ca2+ released from the ER. ② The AST-neuron tripartite synapse: AST regulates synaptic transmission *via* neurotransmitter receptors such as GPCRs located on the AST membrane or gliotransmitters. ③ Proposed patterns of the OPC-neuron synapse: OPCs acts as presynaptic membrane (left) or as postsynaptic domain (right), by which OPCs modulate synaptic transmission.

Notably, as essential components of the CNS glial microenvironment, ASTs and OLGs communicate and interact with each other through Cx-mediated glial networks, and regulate each other in a paracrine manner [10]. Increasing evidence suggests that the interactions between ASTs and OLGs play important roles in modulating various physiological processes in the brain, myelination in particular. For instance, the proliferation and differentiation of OLGs are regulated by factors or extracellular vesicles (EVs) derived from ASTs. In addition, ASTs and OLGs are capable of regulating synaptogenesis and synaptic transmission, suggesting their interaction around synapses (Fig. 1). The interactions between ASTs and OLGs are also involved in the pathogenesis of brain disease, especially demyelinating diseases such as MS, in which both ASTs and OPCs secrete factors to modulate the permeability of the BBB. Moreover, the evidence of OLGs adopting an immune phenotype suggests that ASTs and OLGs co-regulate glia the activation and immune inflammation process (Table 1). In this review, we recapitulate the communication basis of the glial network and mainly focus on the role of AST-OLG interactions in myelin development and related diseases.

**Table 1:**
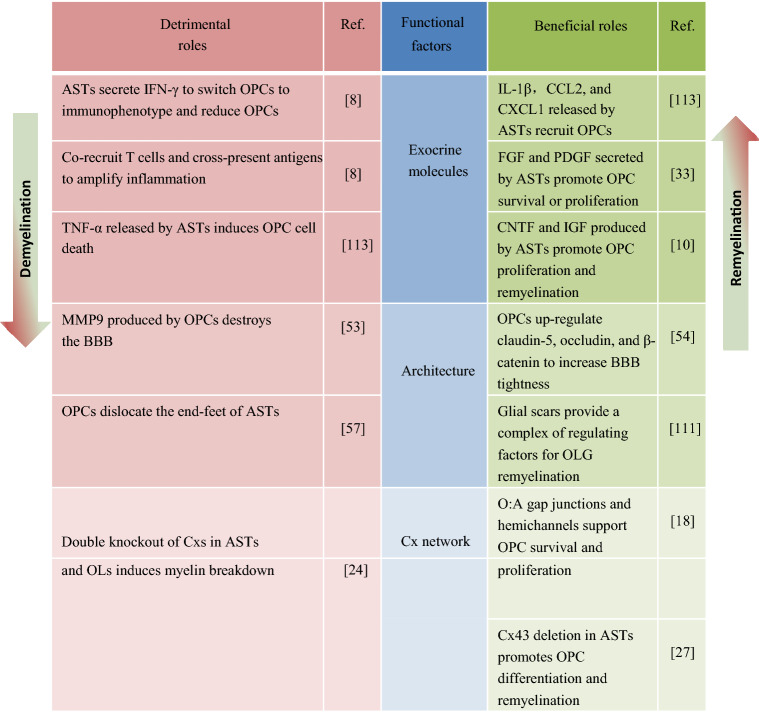
ASTs interplay with OLGs in demyelinating diseases.

## Correlations of ASTs and OLGs during CNS Development

Derived from the same progenitor cells (radial glia/ neural stem cells) in neural epithelia, the first step in the genesis of ASTs and OLGs is specialization, which is determined by a switch of the external glial signals in the niche, such as bone morphogenic protein (BMP) or Notch signaling, activation of which is crucial for AST genesis [11]. Normally, the differentiation of each glial lineage is controlled by a special transcriptional program with the expression of a series of specific genes. For example, in astroglia, glutamate aspartate transporter (GLAST) and glutamine synthetase (GS) are expressed in the immature stage [12], while glial fibrillary acidic protein, S100 β, and Cxs are mainly expressed during or after maturation. During OLG development, platelet-derived growth factor alpha receptor and Neuron-glial antigen 2 (NG2) are mainly expressed in OPCs while myelin basic protein (MBP), myelin proteolipid protein, and myelin oligodendrocyte glycoprotein (MOG) are used to identify OLs [11].

In some cases, such as in culture, OPCs are considered to potentially differentiate into OLs or ASTs [13]. After CNS injury, neurons are rarely replaced, but ASTs and OLGs are highly regenerated through homologous differentiation or replacement by each other. It has been reported that OPCs can produce ASTs by trans-differentiation [14]. Using pedigree tracking in OL-specific presenilin enhancer 2 (Pen-2) knockout mice, it was found that newly-generated ASTs are produced by labeled OPCs. Further studies have shown that deletion of Pen-2 in OPCs promotes the formation of ASTs by the Notch-STAT3 signaling pathway [15]. Recently, it was reported that overexpression of DLX2 by injecting lentivirus (e.g. Lv-gfap-dlx2) into the mouse striatum induces adult ASTs to turn into Achaete-scute homolog 1 (ASCL1) neural progenitor cells (NPCs) within two weeks, and subsequently differentiate into multi-lineage cells such as neurons, ASTs, and OLGs. The reprogramming process is closely similar to endogenous neurogenesis, which requires activation of the Distal-less homeobox (DLX) family and inhibition of the Notch signaling pathway [16]. Although the underlying regulatory mechanism is not clear and the exocrine factors derived from either ASTs or OLGs remain to be identified, it could be speculated that exocrine mechanisms account for this process.

## Infrastructure and Mechanisms of AST-OLG Interactions

### Cx-mediated AST-OLG Communication

In the CNS, a typical feature of glial cells is the strong expression of Cxs, which form gap junctions or hemichannels on the cell surface, enabling the exchange of ions and small molecules between adjacent cells and the direct communication of signals and metabolites [17, 18]. Moreover, these gap junction channels play a pivotal role in the control of the surrounding ionic homeostasis, such as buffering K^+^. Mature ASTs strongly express Cx43 and Cx30 by which they form a complex glial network [19], while Cx47, Cx32, and Cx29 (also known as GJC2, GJB1, and GJC3, respectively) are present in OLs [20, 21]. Besides the homologous gap junction among the same glial type, heteromorphic gap junctions can form between different cell types, including ASTs and OLs (Fig. 1). For instance, OLs are specifically coupled to ASTs through heteromorphic gap junctions composed of Cx47:Cx43 or Cx32:Cx30 (O:A coupling) [22]. Ca^2+^ and glucose can be delivered through these gap junction channels, forming a pan-glial metabolic route between OLGs and ASTs [18, 23, 24]. It has been experimentally demonstrated that Cx47 is more important than Cx30 in the formation of O:A couplings [20]; it is asymmetrical since it shows electrical and metabolic rectification activity [25].

Moreover, hemichannel activity depends on intracellular Ca^2+^ elevation, which is involved in OLG differentiation [26]. It has been found that there are no functional gap junctions between ASTs and OPCs. However, deletion of Cx43 in ASTs inhibits OPC proliferation by decreasing matrix glucose levels without impacting OPC hemichannel properties, which mediate glucose uptake for OPC proliferation [27]. Under pathological conditions, for example, oxidative stress and inflammation in ischemic stroke, the stability of OL gap junctions relies on Cx43 expression in ASTs [28]. A deficiency of Cx43 causes the internalization and degradation of Cx47, hampering the transport of ions and nutrients and spreading inflammatory mediators [29]. As a result, the inflammatory response after loss of glial cells and myelin break-down in ischemia is severely aggravated [30].

The activity of the inflammasome and/or hemichannels of OLGs can be increased by prenatal stress, which is mimicked by urocortin II [31]. Recently, in a mouse model of maternal isolation monitoring early life stress, a decrease in the number of OPCs in the hippocampus was observed, accompanied by developmental disturbance of ASTs and the functional AST network. Furthermore, in the PDGFRαCreER:DTA mouse model, in which OPCs are eliminated by tamoxifen, the morphology of ASTs displayed an atrophic state. Importantly, both expressions of Cx30 and Cx43 was reduced [32], suggesting that OPCs can regulate AST development and network formation.

### Exocrine Regulation of AST-OLG Communication

Both ASTs and OLGs secrete a variety of factors, and regulate each other during development. For example, platelet-derived growth factor AA secreted by ASTs is a trigger or substance that promotes OPC proliferation and maintains their survival by activating the JAK/STAT signal pathway [33]. ASTs also secrete neurotrophic factors such as insulin-like growth factor 1 (IGF-1) and ciliary neurotrophic factor [34], which are essential for OPC differentiation and maturation through the MAPK/ERK signal pathway, while inhibiting oligodendroglial apoptosis *via* PI3K/AKT signaling. In adulthood, IGF-1 has been found to promote OPC differentiation from neural stem cell (NSCs) by inhibiting the BMP signal pathway. Moreover, ASTs release BMPs that prevent OPC differentiation. Therefore, astrocytic factors regulate OL development either positively or negatively (Fig. 1).

Similarly, OPCs can also have a paracrine effect on the development of ASTs. Our recent study, by knockout of Wnt7a/b in OPCs, demonstrated that OPC-derived Wnt7a/b, especially Wnt7b, mediates the regulation and influence on ASTs *via* the Wnt/β-catenin pathway. Then, supplementation of Wnt7a/b can remedy the naive state of ASTs by increasing the expression of Cx43 and Cx30 and enhancing the function of the astroglial network [32].

### Co-regulation of ASTs and OLGs *via* Extracellular Vesicles

EVs are nanometer-sized vesicles secreted by almost all living cells, and the predominant forms are exosomes and microvesicles [35]. Mediated by the containing proteins, lipids, and miRNA, EVs are an alternative mode of intercellular communication for many physiological and pathological functions [36]. Since EVs can even cross the BBB [37], they not only tie intercellular communications throughout the nervous system, but also work as potential biomarkers in the circulation for neurodegenerative diseases such as Alzheimer’s disease [38].

With emerging research, communications between ASTs and OLGs could be achieved by EVs as an option. Recent studies have reported that AST-derived EVs (AEVs) contain fibroblast growth factor-2 and vascular endothelial growth factor (VEGF), which had been confirmed to regulate OPCs in brain development [39]. Critically, AST-derived exosomes have been reported to enhance the chemotaxis of OPCs, improving their differentiation and migration under ischemia *in vitro* and inhibiting their proliferation under severe hypoxia [40]. In addition, AEVs with aging phenotypes have negative effects on OL maturation [41]. Moreover, ASTs can augment the production of exosomes by OPCs *via* ITGB4-mediated cell adhesion and thus stimulate OPC proliferation [42]. Therefore, the secretion of EVs seems likely to be the result of the interaction between OPCs and ASTs.

Subsequently, OLs-derived EVs (OEVs) were also discovered. They generally store myelin components, such as CNPase (2’,3’-cyclic-nucleotide 3’-phosphodiesterase), MBP, MOG, myelin-associated glycoprotein, cholesterol, and sphingolipids, and thus contribute to the regeneration and maintenance of myelin [43], increasing action potential promoting axonal transport [44]. Besides, intercellular delivery of OEVs containing SIRT2 are critical for axonal energy enhancement [45]. Similarly, AEVs loaded with synapsin-1 foster axonal growth, the functional maturation of synapses, and synaptic plasticity. The release of EVs by both may have special regulation patterns in synapses, but the exact mechanism remains to be explored. Moreover, OEVs can aid neuronal resistance to ischemic stress *in vitro* by transferring superoxide dismutase and catalase [46], while Apo-D in AEVs has been found to resist oxidative stress and be essential for anti-aging and the prevention of neurodegenerative diseases. Recently, EVs have been shown to be loaded with immune factors and complement, mediating immune regulation [39, 47].

Notably, a recent study pointed out that conditional knockdown of dicer, an essential miRNA synthetic enzyme in ASTs, inhibits OPC differentiation and delays remyelination in the lysophosphatidyl-induced demyelination mouse model [48], suggested an important role of AST-derived miRNAs in OPC differentiation. It has been found that miR-302/367 induces the conversion of astrocytes to OLGs and enhances myelin repair *in vivo* [49]. Given that AST-derived EVs (AEVs) promote OPC migration and differentiation under severe hypoxic conditions [40], it is possible that miRNA-enriched AEVs may be involved in the regulation of OPC differentiation; this requires further investigation.

## AST-OLG Interactions and BBB Integrity

The BBB is a physical protective interface composed of multiple cell-types. It maintains the immune privilege and dynamic stability of the CNS. As an important component of the BBB, ASTs play a vital role in the maintenance of its integrity. In recent years, OPCs have attracted attention due to their close positional relationship with the BBB. During CNS development, OPCs use the intracephalic vascular system as a scaffold for migration [50], suggesting that they may have close contact with ASTs. By adding OPC-conditional media to endothelial cell cultures, subsequent pharmacological experiments demonstrated their ability to activate the MEK/ERK signaling pathway and enhance the expression of tight junction proteins *in vitro* [10, 51], suggesting an important role of OPCs in the permeability of the BBB. Moreover, OPC-specific transforming growth factor β (TGF-β)-deficient mice show cerebral hemorrhage and BBB damage, suggesting that OPC-derived factors regulate BBB integrity [52]. Under pathological conditions, such as demyelination in mice induced by long-term cerebral hypoperfusion stress, matrix metalloproteinase-9 secreted by OPCs induces early BBB destruction and inflammatory infiltration [53]. In the ischemic stroke model of transient arterial occlusion (90 min), OPC transplantation can activate the Wnt/β-catenin pathway to protect the BBB during the acute phase of ischemic stroke and promote the recovery of neurological function. Further studies have demonstrated that OPCs can rescue BBB leakage by increasing the expression of claudin-5, occludin, and β-catenin in endothelia [54].

Because of their close relationship with the vascular system, OPCs may not only participate in the pathogenesis of the BBB, but also be victims of its destruction. For example, after BBB breakdown, the increased vascular permeability leads to the leakage of plasma proteins, destroying the original micro-environmental homeostasis. As a consequence, the coagulation factor fibrinogen induces the activation of BMP signaling in OPCs and thus inhibits their differentiation and remyelination [10]. Indeed, at the beginning of demyelination, such as in experimental autoimmune encephalomyelitis (EAE), a mouse model of MS, it has been reported that tumor necrosis factor (TNF) secreted by ASTs or endothelial cells may cooperate with TNF receptors on the surface of OLs to damage the BBB [55]. This is accompanied by the upregulation of hypoxia-inducible factor-1 directly produced by activated ASTs and microglia, which boost VEGF and interleukin (IL)-1 and further aggravates the BBB permeability in EAE [56]. Dysfunctional Wnt signaling in MS can result in OPC aggregation along the blood vessels, which may interfere with AST end-feet on the vascular surface [57], leading to altered vascular permeability and the related inflammatory response. Thus the interaction between ASTs and OPCs is important in regulating the integrity of the BBB (Fig. 1).

Interestingly, deficits in Cxs can also induce BBB injury [58]. Compared with normal mice, Cx47-KO mice are more susceptible to EAE and exhibit more intense pathologies, including destruction of the blood-spinal cord barrier, infiltration of inflammatory cells into spinal cord tissue, and apoptosis of OLs [59, 60]. Thus, the Cx-mediated AST-OLG networks are also involved in the regulation of BBB integrity.

## AST-OLG Interactions in Nourishing Neurons

### Modes of Neuronal Energy Support

In the human brain, glucose is the major energy source for neuronal activity; alternative energy sources include ketone bodies, lactate, fatty acids, and amino-acids. As neurons consume high energy for neural transmission but lack glycogen storage, so they rely on glial cells for glucose acquisition [61, 62]. ASTs, as the main glycogen storage in the brain, provide nutritional support to neurons by taking up glucose from blood vessels through the glucose transporter GLUT1 and also take up various metabolites released by neurons, such as lactate and glutamate [63]. Lactate is not only an effective fuel for neuronal activity [64], but also acts as an intercellular messenger to shuttle between ASTs and neurons [65]. In addition, ASTs play an essential role in maintaining glutamate homeostasis by recycling glutamine, an intermediate of the tricarboxylic acid cycle critical for generating glutamate, back to neurons.

Besides transporting glucose to neurons, ASTs are the only type of cells in the brain that oxidize fatty acids and produce ketone bodies to support energy for neurons in hypoglycemia or starvation states [1]. It should be noted that ketone bodies, as with lactate, can cross the BBB *via* MCTs in endothelial cells and ASTs. During long-term fasting (5–6 weeks) in the obese patients, ketone body levels in the brain increase significantly, providing almost 60% of the brain's energy, therefore ketone replaces glucose as the primary fuel [66].

Compared to ASTs and neurons, OLGs show the highest glucose utilization because of their requirement of lipid synthesis for myelination [67]. Due to the physical isolation of the myelin sheath, OLGs support axons by supplying energy substrates. In terms of energy substrates, OLs actually prefer lactate to glucose as the substrate for myelin production, as lactate produces more myelin in brain slices than glucose-treated slices. It has been reported that OLs use three times more lactate than ASTs and neurons [68]. Some experiments have shown that OLGs express MCT1, a selective transporter of lactate, which provides lactate to support axon integrity. It has been found that conditional knockout of MCT1 in OLs leads to myelin dysfunction and axonal degeneration [69], and impairment of MCT1-mediated lactate transport from OLs to axons is considered to contribute to the pathogenesis of amyotrophic lateral sclerosis [4].

### AST-OLG Cooperation in the Lactate Shuttle

In the CNS, the cellular distribution and physiological characteristics of MCTs suggest an energy correlation between different cell types by the lactate shuttle [70]. MCT1 is mainly expressed in OLGs and a few specific neurons [71], MCT2 is chiefly expressed in neurons, and MCT4 is principally expressed in ASTs. According to previous studies, ASTs transfer substrates for energy metabolism directly to OLs through heterotypic gap junctions. And it has been speculated that mature OLs provide lactate to axons through MCT1 as energy during high metabolic activity such as myelination, while lactate can also be shuttled back and forth between neurons and ASTs through MCT2 and MCT4 [72]. Some studies have shown that, in the middle cerebral artery occlusion mouse model, after ischemic reperfusion, the expression of MCT1 in the striatum is significantly up-regulated [73]. The vulnerability of OLGs to metabolic stress is related to their distinct MCT1 expression profiles of, the mild hypoxia-glucose deprivation caused by ischemia triggers the upregulation of MCT1 in OPCs to adapt to stress [74], and this enhances the transport of lactate from ASTs and blood to redistribute energy substrates. Moreover, due to the hypermetabolic demand for myelin generation, OLGs are the cells with the highest iron levels in the brain [75].

Since ASTs occupy an important position in obtaining nutrients including iron from circulating blood, upregulation of iron importers such as transferrin receptor, divalent metal transporter 1, and ZIP14 in ASTs in the EAE model was considered to favor iron supply for OLGs. Meanwhile, as the ferroportin-ceruloplasmin system is responsible for cellular iron efflux, the expression of ferroportin (SLC40A1) in ASTs suggests their important role in iron homeostasis [76, 77],which is relevant to OLG maturation and myelination [78] . Nevertheless, OLGs show relatively low glycolytic enzyme expression, and they communicate with ASTs *via* gap junctions, allowing the transfer of lactate and other metabolites between the two kinds of cells [79]. Therefore AST-OLG cooperation in the lactate shuttle is an important pathway for neuronal energy support (Fig. 1).

## AST-OLG Interactions in Neural Plasticity

### The AST-Neuron Tripartite Synapse

The synapse is the structural unit that mediates signal transmission between neurons. However, neuronal synapses are not only composed of presynaptic and postsynaptic neurons, but also are connected by AST processes in many cases to form tripartite synapses [3]. Similar to neurons, ASTs express a variety of neurotransmitter receptors in the presynaptic or postsynaptic membranes, such as receptors for glutamate, gamma-aminobutyric acid (GABA), endogenous cannabinoids, dopamine (DA), 5-hydroxytryptamine, ATP/adenosine, acetylcholine, and opioids. Many of the neurotransmitter receptors of ASTs are G protein-coupled receptors (GPCRs) [80]. Once activated by neurotransmitters released from the presynaptic membrane, the elevation of intracellular Ca^2+^ causes ASTs to release signaling molecules, namely gliotransmitters, which in turn modulate synaptic transmission, either excitatory or inhibitory. The gliotransmitters glutamate, GABA, D-serine, and ATP may differently participate in long-term potentiation (LTP), long-term depression, and heterosynaptic facilitation/depression [81].

It has been shown that purinergic signals such as ATP and adenosine play important roles in regulating synaptic activity and function. Purinergic receptors are classified as P1 (adenosine receptors) and P2 (ATP receptors), both of which are involved in neuron-ASTs interactions. Numerous studies have shown that synaptic activity causes ATP released from ASTs to induce homosynaptic and/or heterosynaptic inhibition. Extracellular adenosine levels are largely dependent on ATP release by ASTs. Adenosine inhibits synaptic transmission by activating A1 receptors while activation of the A2A receptor enhances this process [82]. It is important to note that, due to the difference in the distribution of presynaptic receptors, AST-derived ATP or adenosine can have different effects on different neurons or neural circuits. For example, hippocampal ATP/adenosine derived from ASTs down-regulate excitatory synaptic transmission by activating presynaptic adenosine A1 receptors and enhance inhibitory synaptic transmission by activating postsynaptic P2Y1 receptors [83], thereby effectively down-regulating the excitability of the entire hippocampal neural circuit.

Moreover, D-serine released by ASTs can act as an endogenous co-agonist of postsynaptic N-methyl D-aspartate receptors (NMDARs), enhancing NMDAR activity. For example, activation of α7n-acetylcholine receptors in ASTs results in the release of D-serine, which enhances postsynaptic NMDAR activity and fear memory [84]. Activation of cannabinoid receptor 1 (CB1) in ASTs also increases Ca^2+^ activity and releases D-serine, which in turn activates NMDARs to enhance hippocampal-dependent object recognition memory. CB1-knockout in ASTs inhibits LTP in the hippocampus and reduces object recognition memory [85], effects that can be reversed by exogenous D-serine supplementation. In addition, the K^+^ channels Kir4.1, mGluR3, and mGluR5 have been shown to monitor and regulate synaptic function, thus actively controlling synaptic transmission [86].

Although the regulatory mechanism remains unclear, IP3R2 (receptor for inositol 1,4,5-trisphosphate) has been considered the main regulator of GPCR-mediated Ca^2+^ mobilization in ASTs. Consistent with this, in IP3R2 knockout mice, neurotransmitters have no impact on intracellular Ca^2+^ in ASTs [87].

### Astrocytic Factors Regulating Synaptogenesis

Although most neurons are generated in the embryonic stage, synapses are not formed until birth and massively increase at the end of the first postnatal week after AST differentiation in mice. Increasing evidence indicates that signals derived from ASTs are essential for synaptogenesis and neural circuit formation, functional maturity, and improvement [88]. Experiments have shown that rodent neurons form very few synapses *in vitro*, while co-culture with ASTs greatly increases the number of synapses [89]. ASTs secrete many cytokines or gliotransmitters to regulate synapse formation, such as BDNF, TNF-α, TGF-β, thrombospondins (TSPs), glypicans, and SPARCL1. For example, TSP1/2 and SPARCL1 (secreted protein acidic and rich in cysteine)-like 1)/Hevin control the formation of glutamatergic synapses [90]. ASTs also produce a synaptic Hevin antagonist called SPARC, which negatively regulates the number and function of synapses. In addition, mediated by Wnt signaling, glial cells can affect synapse formation, pruning, and maturation by accumulating glutamate receptors in excitatory synapses [91].

Some experiments have shown that TSP promotes remodeling of the actin cytoskeleton during initial contact of synapses by activating the Rac1 pathway. With the secretion of TSP, ASTs also release the innate immune molecule pentraxin3, which promotes the maturation of excitatory synapses by accumulating AMPARs (α-amino-3-hydroxy-5-methyl-4-isoxazolepropionic acid receptors) in the postsynaptic membrane [92].

Moreover, to prevent excessive and inappropriate synapses, ASTs regulate synaptic elimination [93]. It has been reported that ASTs directly eliminate excess synapses in the developing brain through the MEGF10 and MERTK phagocytic pathways [94]. ASTs also indirectly regulate synaptic elimination by secreting TGF-β, which mediates synaptic elimination by microglia [95].

### OLGs Modulate Synaptogenesis and Neural Transmission

Besides forming the myelin sheath around axons, OLGs also interact with neurons and affect neural networks at the synaptic level. It has been shown that OPCs express an array of receptors (AMPARs, NMPARs, and GABARs), which are endowed with the capacity to respond to neuronal activity and thus regulate the survival, proliferation, migration, differentiation, and myelination of OLGs. However, the expression and function vary among these receptors. For example, following the discovery of excitatory synaptic transmission between neurons and NG2+ OPCs [96], the expression of AMPARs has been found in OLGs and changes with OLG development. AMPARs are highly expressed in OPCs, and down-regulate (~12-fold) upon OPC differentiation into mature OLs [97]. Sufficient evidence has shown that activation of AMPARs inhibits OPC proliferation, while blocking AMPAR activity impairs the morphological development of OPCs and promotes OPC proliferation and differentiation [98].

In addition to the AMPAR, another key ionotropic receptor mediating glutamate transmission is the NMDAR. NMDAR currents peak during the critical time of myelination, decline with sexual maturity, and completely disappear at 9 months in mice [97, 99]. The role of NMDARs in neuron-OPC synapses is controversial. Some experiments have demonstrated that NMDARs are not required for OPC proliferation and myelination, and the presence of NMDA in these synapses only accounts for the AMPAR-dependent signals in OPCs [99].

What is more, GABAA receptors were first confirmed in explant cultures of spinal cord [100], then GABAA receptor-evoked depolarization in OPCs has been described in different CNS regions. The expression of GABAA receptors is down-regulated during the differentiation process. It has been suggested that GABA plays a central role in regulating OPC proliferation, differentiation, and myelination. Stimulating GABA activity in OPCs inhibits cell proliferation but promotes differentiation, while sensitivity to GABA is largely reduced in mature OLs [101]. To date, the understanding of OPCs has been limited to passively receiving neuron signal transmission in neuron-OPC synapses. However, a recent report demonstrated that NG2+ OPCs form presynaptic membranes with neurons in hippocampus. Photo-stimulating NG2 glia functionally drives GABA release and enhances inhibitory synaptic transmission to proximal interneurons, which may be correlated with anxiety-like behavior in mice [102]. Moreover, in a recent study, conditional ablation of Nogo-A in OLs increases the density and length of dendritic spines in motor cortical pyramidal cells [103], suggesting that the OL-specific protein Nogo-A is a regulator of events in synaptic refinement.

On the other hand, the continuous production of OPCs in the adult brain can form new myelin sheaths, which can wrap and produce a new sheath, affect the thickness of the sheath, and regulate the conduction velocity of the axon. To examine the functional significance of myelination on white matter injury (WMI), an emerging study by knocking out Olig2 (loss-of-function) or M1R (gain-of-function) in OPCs, demonstrated that hypo-myelination results in the loss of excitatory synapses and functional deficits. Enhancing myelination rescues synaptic deficits and improves motor behavior in mice after chronic hypoxia [5], suggested that myelination may facilitate excitatory presynaptic innervation.

### A Speculation: AST-OLG Interplay at the Synaptic Level

Although ASTs and OLGs are involved in the formation of and transmission by synapses by sharing the same receptors, signals and transmitters, such as AMPARs and NMDARs, no direct evidence has ever shown the interaction of both cells at the synaptic level. Given the architecture of AST-neuron tripartite synapses, and that OPCs can form GABAergic presynaptic membrane with neurons, we boldly speculate that OPCs may modulate synaptic transmission by neurotransmitter receptors or gliotransmitters the same as the AST-neuron tripartite synapse. Is it possible that OPCs surround the AST-neuron tripartite synapse with their processes to form a kind of tetragonal synapse? These interesting ideas are worthy of further study. Functionally, both glial cells were found to be able to regulate synaptic formation or plasticity in learning and memory. During this process, the communication between ASTs and OLGs, such as the glial network mediating material delivery, and the regulatory factors secreted by each cell, are likely to further promote or inhibit the formation or function of synapses. For example, the release of L-lactate by ASTs has been found necessary for long-term memory [104], while lactate metabolism involves the interactions between the two types of glial cells in previous studies [105]. In addition, gliotransmitters around the AST-neuron tripartite synapse, such as adenosine, are involved in regulating OPC development. It has been suggested that adenosine not only inhibits the proliferation of OPCs, but also stimulates the migration and differentiation of OPCs and promotes myelin formation [106]. Thus an interaction between ASTs and OLGs may exist and play an important role in synaptogenesis and plasticity (Fig. 1).

## AST-OLG Interactions in Neuroinflammation

### AST Activation in Neuroinflammation

The CNS has been considered to be immune exempted due to its unique anatomical features, including the relative absence of lymphoid drainage and specific antigen-presenting cells such as dendritic cells, the absence of secondary lymphoid organs, and the presence of mechanical barriers such as the BBB that limit the exchange of immune cells and molecules [107]. Therefore, the immune defense mechanism mainly relies on the natural immune cells residing in CNS, microglia and ASTs, which respond quickly to all kinds of insults, such as ischemia-hypoxia, injury, and infection. Activated by membrane receptors including toll-like receptors [108], purinergic receptors, and triggering receptors expressed on myeloid cells 2 (TREM2), microglia can either recruit peripheral monocytes and lymphocytes by releasing chemokines, or induce AST activation by releasing cytokines. Activation of ASTs is a common response in many pathophysiological conditions. Active ASTs are characterized by cell body hypertrophy, thickening of processes, increased branching, eosinophilia, and release a series of factors, such as cytokines (lipocalin 2, IL-1β, and TNF-α) and neurotrophic factors (BDNF and VEGF). These cytokines play either neuroprotective or neurotoxic role, ultimately triggering inflammatory responses or exacerbating CNS injury.

Taking MS as an example, microglia and ASTs have been considered to be the key players in neuroinflammation to induce OL damage and demyelination in the initial phase [109]. While the role of glial scars, one of the stuctures responsible for neural damage (such as demyelination), is controversial (Tabe1). Previously, glial scars were thought to hinder OPC survival and migration, thus inhibiting remyelination [110]. Recently, a study indicated that glial scars do not have a rigid border but rather provides a complex of regulating factors for OLG remyelination [111]. Nevertheless, recent studies have demonstrated that OPCs can transform into a disease-specific cellular state characterized by the activation of genes previously thought to be unique to immune cells, thus providing a new perspective for the study of neuron-immune diseases such as MS [8].

### OLGs Adopt an Immunophenotype in MS

MS is an autoimmune demyelinating disease in which the immune inflammatory response is activated to attack myelin and OLs [112]. Myelin repair occurs in all stages of MS, and often coexists with demyelination. However, a number of studies have shown that OPCs are abundant in the injured areas but fail to differentiate due to local inflammation [109], which may account for the bad outcome of treatment.

Recent analysis by flow cytometry and RT-qPCR of OLGs in EAE have mice revealed that EAE-specific OLGs express genes involved in antigen processing and presentation including major histocompatibility complex class I and II (MHC-I and -II), and interferon response genes, including toll-like receptor 3 and members of the serpina gene family [113]. These disease-specific OLGs or the immunological OPC state are also present in MS brain tissue. For instance, in the lesion areas, OLGs express the immunoproteasome subunit PSMB8, which is not found in normal white matter [8].

To investigate how neuroinflammation influences OLGs, researchers carried out a fate-tracing study in a mouse model of inflammatory demyelination. The study revealed that OPC differentiation is inhibited by IFN-γ released by both effector T cells and ASTs [8]. The absolute number of OPCs is significantly reduced, while immunoproteasomes and MHC class I are induced in the remaining OPCs under induction by IFN-γ. Moreover, *in vitro* studies have shown that OPCs are capable of phagocytosis and MHC-II-expressing OPCs activate memory and effector CD4-positive T cells. In EAE, OLGs co-present MHC class I-restricted MBP with Tip-dendritic cells to cytotoxic CD8 T cells [113]. In a newly-published study, the RNA sequencing of cultured OPCs exposed to inflammatory cytokines (IFN-γ, IL-1β, and TNF) revealed that OPCs are able to generate chemokines to recruit and activate microglia, and the transformation of OPCs toward an immune phenotype seems to be mediated by TNFR2 signaling [114], undermining their ability to proliferate and differentiate. Moreover, OPCs can facilitate the migration of microglia by releasing chemokines such as CCL2 [115], or participate in immunoregulation through secreting CCL5, CX3CL1, and CXCL10 [116]. Therefore, OLs and OPCs are not passive targets, but instead, they can co-operate with microglia and ASTs to perpetuate the autoimmune response in MS [117].

## Concluding Remarks

As two types of non-excitable nerve cells, ASTs and OLGs have been found to be functionally involved in brain activity and the pathogenesis of brain diseases in recent studies. In particular, these two highly homologous glial cells function closely *via* a Cx-mediated network and exocrine mechanisms. Nevertheless, the detailed patterns are not fully understood, and their interaction is more complicated because of the newly-proposed concept of glial heterogeneity [118]. Hence, we pose the following questions. Are the glial networks between ASTs and OLGs consistent or diverse in subtypes and in distinct brain regions? Are those glial networks formed by different subtypes of ASTs or OLGs affected by diseases? While biochemical molecules mediate the interaction between the two glial cells, the quantities and corresponding receptors of the related molecules released by those subtypes of glial cells in various brain regions remain undefined. These complex patterns may underlie the mechanism by which glial cells precisely modulate neural transduction of CNS.

It is desirable to explore the spatio-temporal regulation patterns between ASTs and OLGs, as well as their modulatory effect on specific neural circuits in further studies. New technologies such as *in vivo* Ca^2+^ imaging, cell fate tracing, viral loop tracking, chemogenetic and optogenetic tools, and 3D organic culture may uncover the new properties of glial interactions [119]. Disclosing the regulatory mechanisms underlying AST-OLG interaction may provide novel insights into therapeutic strategies for related brain diseases.
